# Frequency and diversity of small cryptic plasmids in the genus *Rahnella*

**DOI:** 10.1186/1471-2180-10-56

**Published:** 2010-02-19

**Authors:** Wilfried Rozhon, Elena Petutschnig, Mamoona Khan, David K Summers, Brigitte Poppenberger

**Affiliations:** 1 Max F Perutz Laboratories, University of Vienna, Dr Bohrgasse 9, 1030 Vienna, Austria; 2 Albrecht-von-Haller-Institute of Plant Sciences, Georg-August-University Goettingen, Untere Karspuele 2, 37073 Goettingen, Germany; 3 Department of Genetics, University of Cambridge, Downing Street, Cambridge CB2 3EH, UK

## Abstract

**Background:**

*Rahnella *is a widely distributed genus belonging to the Enterobacteriaceae and frequently present on vegetables. Although *Rahnella *has interesting agro-economical and industrial properties and several strains possess antibiotic resistances and toxin genes which might spread within microbial communities, little is known about plasmids of this genus. Thus, we isolated a number of *Rahnella *strains and investigated their complements of small plasmids.

**Results:**

In total 53 strains were investigated and 11 plasmids observed. Seven belonged to the ColE1 family; one was ColE2-like and three shared homology to rolling circle plasmids. One of them belonged to the pC194/pUB110 family and two showed similarity to poorly characterised plasmid groups. The G+C content of two rolling circle plasmids deviated considerably from that of *Rahnella*, indicating that their usual hosts might belong to other genera. Most ColE1-like plasmids formed a subgroup within the ColE1 family that seems to be fairly specific for *Rahnella*. Intriguingly, the multimer resolution sites of all ColE1-like plasmids had the same orientation with respect to the origin of replication. This arrangement might be necessary to prevent inappropriate synthesis of a small regulatory RNA that regulates cell division. Although the ColE1-like plasmids did not possess any mobilisation system, they shared large parts with high sequence identity in coding and non-coding regions. In addition, highly homologous regions of plasmids isolated from *Rahnella *and the chromosomes of *Erwinia tasmaniensis *and *Photorhabdus luminescens *could be identified.

**Conclusions:**

For the genus *Rahnella *we observed plasmid-containing isolates at a frequency of 19%, which is in the average range for Enterobacteriaceae. These plasmids belonged to diffent groups with members of the ColE1-family most frequently found. Regions of striking sequence homology of plasmids and bacterial chromosomes highlight the importance of plasmids for lateral gene transfer (including chromosomal sequences) to distinct genera.

## Background

*Rahnella*, a genus of the Enterobacteriaceae, is commonly found in the rhizosphere [1,2] and phyllosphere [3], seeds [4], fruits [5,6], water [7] and intestinal tracts of herbivores including snails, slugs, and even American mastodon remains [8,9]. In addition, *Rahnella *strains have been isolated from the extreme environment of uranium and nitric acid contaminated soil adjacent to disposal ponds of the DOE Field Research Centre in the Oak Ridge National Laboratory Reservation in Tennessee [10]. The genus *Rahnella *comprises three closely related species that cannot be phenotypically differentiated: *Rahnella aquatilis*, *Rahnella *genomospecies 2 and *Rahnella *genomospecies 3 [8].

In recent years interest in certain *Rahnella *strains increased because of their remarkable properties. *Rahnella *might be useful for control of plant pathogens like *Erwinia amylovora *causing fire blight of apple trees or *Xanthomonas campestris*, the causal agent of black rot [11,12]. Several strains seem to improve plant nutrition, as they are able to fix nitrogen [2] and to solubilise hydroxyapatite, thus converting phosphate to a plant utilisable form [13]. The production of polysaccharides, especially levan and lactan, by different *Rahnella *isolates is intensively studied, because these macromolecules have ideal properties for industrial applications [14-16]. Some reports have described *Rahnella *as an opportunistic human pathogen but infections with *Rahnella *are usually limited to immunocompromised patients and recovery is rapid [17-19]. However, antibiotic resistances and enterotoxins encoded by several strains [20-22] might spread within microbial communities. Thus, an improved understanding of mobile genetic elements of *Rahnella *is crucial to assess the potential of lateral gene transfer to other species including human pathogens. Nevertheless, although *Rahnella *is widely distributed and frequent on vegetables and therefore likely to be routinely present in the human diet, little is known about plasmids of this genus. So far only one *Rahnella *plasmid, pHW15, has been characterised [6]. pHW15 belongs to the ColE1 family, is non-mobile and stably maintained even in the absence of selective pressure. To gain insights into the frequency, diversity and evolution of small (less than 15 kb) *Rahnella *plasmids, we isolated strains from different geographic origins and sample materials. Most plasmids belonged to the ColE1 family but we also found members of other groups, including plasmids replicating by the rolling circle mechanism. In addition, sequence analysis provided evidence for lateral gene transfer within *Rahnella *as well as between *Rahnella *and other genera.

## Results and Discussion

### Isolation of strains, screening for plasmids and cloning

**Figure 1:**
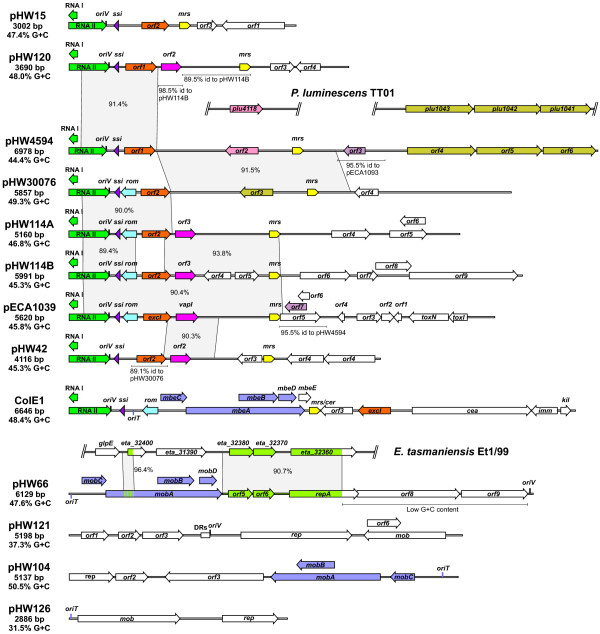
**Maps of plasmids and homologous sequences**. Same colours indicate homologous genes, operons or genetic elements (*mrs*, *ssi*). Larger regions exhibiting more than 85% sequence identity at the DNA level are marked with grey areas or are indicated below the sequence. Nucleotide sequence identities are given in percent. Replication and transfer origins are shown above the DNA when they are located on the sense strand and below if they are placed on the antisense strand. The plasmids pECA1039 and ColE1 as well as parts of the chromosomes from *P. luminescens *TT01 and *E. tasmaniensis *Et1/99 are shown for comparison. Abbreviations: DRs, direct repeats; *mrs*, multimer resolution sites; *oriT*, origin of transfer; *oriV*, origin of replication; *ssi*, single strand initiation site.

Forty five *Rahnella *strains were isolated from vegetables obtained from supermarkets or sampled from fields. Isolates from the same sample were only included in the collection if they differed in at least one biochemical characteristic or the partial 16S rRNA gene sequence to avoid multiple sampling of the same strain. This collection was complemented by 6 strains obtained from culture collections and two strains that had been previously investigated for plasmid content (Table 1). Thus, in total 53 strains were included in this study and 10 of them (19%) contained small plasmids, as revealed by DNA isolation and subsequent gel electrophoresis. Nine of these strains contained one plasmid and one of them had two. Therefore, 10 novel plasmids were detected in addition to pHW15. Their sizes ranged from 2.9 to 7.0 kb, which is typical for small plasmids from enterobacteria [23]. The method we used for detection of plasmid DNA preferentially selects for small plasmids (below 20 - 30 kb) rather than large DNA molecules. Thus, the presence of large plasmids in the investigated strains cannot be excluded. Cloning and sequencing of the isolated plasmids revealed that the majority of them (7 of 11; 64%) belonged to the ColE1 group (plasmids pHW15 to pHW42, Fig. 1). In addition, one ColE2-like plasmid (pHW66) was isolated. The three remaining plasmids (pHW121, pHW104 and pHW126) are likely to replicate by the rolling circle mechanism. pHW121 belonged to the well-described pC194/pUB110 family, while pHW104 and pHW126 showed homology to different groups of poorly characterised plasmids.

**Table 1:** Strains used in this study

Strain^a^	Genomic G+C content^b^	Plasmid	Source	Year of isolation	Geographic region	Reference
DSM 4594^Tc^	51.7 ± 0.5	pHW4594	Water	Before 1976	France	[60]
DSM 30076	51.4 ± 0.4	pHW30076	Chicken	1984 - 1988	Not given	[8]
DSM 30078		-	Minced meat	1984 - 1988	Not given	[8]
CCUG 21213^d^		-	Human burn	1984 - 1988	USA	[8]
CCUG 48021^e^		-	Snail, intestinal content	1984 - 1988	Germany	[8]
CCUG 48023^f^		-	Human blood	1984 - 1988	Germany	[8]
WMR15	51.9 ± 0.9^g^	pHW15	Pear, fruit	2000	Austria	[6]
WMR39		-	Carrot, root	2002	Austria	This study
WMR41		-	Carrot, root	2002	Austria	This study
WMR42	51.5 ± 0.2	pHW42	Carrot, root	2002	Spain	This study
WMR52		-	Carrot, root	2002	Austria	This study
WMR58	51.8 ± 0.7^g^	-	Carrot, root	2002	Austria	[6]
WMR59		-	Leek, root	2002	Austria	This study
WMR60		-	Leek, root	2002	Austria	This study
WMR65		-	Spring onion, root	2002	Austria	This study
WMR66	51.8 ± 0.6	pHW66	Spring onion, root	2002	Austria	This study
WMR67		-	Celery, root	2002	Austria	This study
WMR70		-	Celery, root	2002	Austria	This study
WMR75		-	Sugar beet, root	2002	Austria, Lower Austria	This study
WMR76		-	Sugar beet, root	2002	Austria, Lower Austria	This study
WMR77		-	Yellow carrot, root	2002	Austria	This study
WMR79		-	Yellow carrot, root	2002	Austria	This study
WMR81		-	Yellow carrot, root	2002	Austria	This study
WMR82		-	Parsley, root	2002	Austria	This study
WMR83		-	Parsley, root	2002	Austria	This study
WMR84		-	Beetroot, root	2002	Austria	This study
WMR86		-	Beetroot, root	2002	Austria	This study
WMR87		-	Horseradish, root	2002	Austria	This study
WMR88		-	Horseradish, root	2002	Austria	This study
WMR93		-	Radish, root	2002	Austria	This study
WMR94		-	Carrot, root	2002	Spain, Gran Canaria	This study
WMR95		-	Carrot, root	2002	Spain, Gran Canaria	This study
WMR97		-	Carrot, root	2002	Spain, Gran Canaria	This study
WMR98		-	Carrot, root	2002	Spain, Gran Canaria	This study
WMR100		-	Celery, root	2003	Germany	This study
WMR102		-	Carrot, root	2003	Germany	This study
WMR104	52.2 ± 0.3	pHW104	Carrot, root	2003	Germany	This study
WMR105		-	Carrot, root	2003	Germany	This study
WMR106		-	Carrot, root	2003	Italy	This study
WMR107		-	Carrot, root	2003	Italy	This study
WMR108		-	Carrot, root	2003	Italy	This study
WMR109		-	Potato, tuber	2003	Egypt	This study
WMR113		-	Leek, root	2003	Belgium	This study
WMR114	51.3 ± 0.2	pHW114A+B	Leek, root	2003	Belgium	This study
WMR120	52.6 ± 0.5	pHW120	Carrot, root	2005	Spain, Tenerife	This study
WMR121	52.5 ± 0.5	pHW121	Carrot, root	2005	Spain, Tenerife	This study
WMR126	52.2 ± 0.1	pHW126	Carrot, root	2006	Albania	This study
WMR128		-	Carrot, root	2006	Croatia, Dubrovnik	This study
WMR138		-	Carrot, root	2006	Spain, La Palma	This study
WMR140		-	Carrot, root	2006	Spain, La Palma	This study
WMR141		-	Carrot, root	2007	Portugal, Madeira	This study
WMR143		-	Carrot, root	2007	Portugal, Madeira	This study
WMR144		-	Carrot, root	2007	Portugal, Madeira	This study

^a ^DSM strains were obtained from the Deutsche Sammlung von Mikroorganismen und Zellkulturen GmbH, Germany. CCUG strains were obtained from the Culture Collection, University Göteborg, Sweden.

^b ^Means and standard deviations of the mol% G+C contents were calculated from at least three independent measurements.

^c ^Synonyms: CCUG 14185^T^, ATCC 33071^T^, CUETM 77-115^T^, MCCM 01700^T^, CDC 1327-79^T^.

^d ^Synonyms: CDC 658-79, MCCM 01948.

^e ^Synonyms: SM S7/1-576, CDC 4402-96.

^f ^Synonyms: SM Bonn 7, CDC 4418-96.

^g ^Taken from [6].

### ColE1-like plasmids

**Figure 2:**
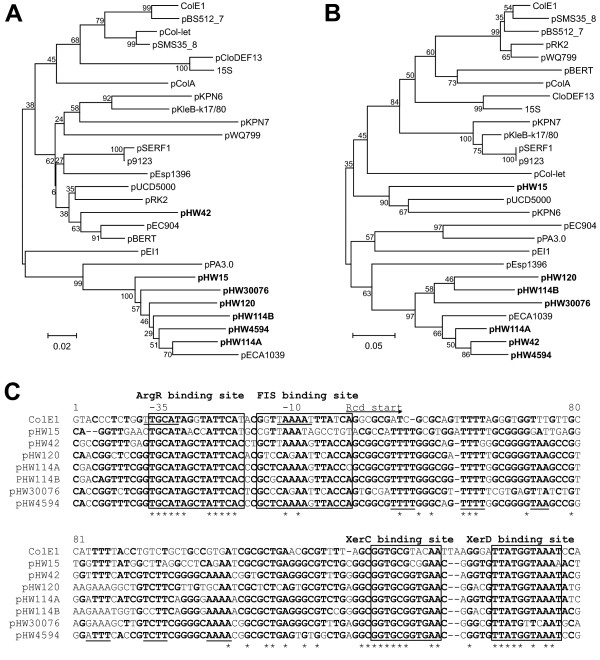
**The ColE1-like plasmids of *Rahnella *form a sub-family**. Phylogenetic trees were constructed based on RNA II (A) or the *mrs *(B). In both trees most ColE1-like plasmids isolated from *Rahnella *(shown in bold letters) formed a cluster with pECA1039, a plasmid of *Pectobacterium artrosepticum*. (C) Alignment of the multimer resolution sites. The ArgR, FIS, XerC and XerD binding sites are boxed and conserved A-T stretches responsible for DNA bending are underlined. The -10 and -35 boxes of the ColE1 P_*cer *_promoter are underlined and the start of the Rcd coding region is indicated by an arrow. Nucleotides conserved in at least 50% of the sequences are shown in bold and invariant sites are marked with an asterisk.

The replication regions of the ColE1-like plasmids showed the typical elements: RNA I, RNA II, a single strand initiation site (*ssi*) and a *terH *sequence for termination of lagging-strand synthesis. Phylogenetic analysis based on the RNA II sequence revealed that pHW15, pHW120, pHW114A, pHW114B, pHW30076 and pHW4594 represented a subgroup within the ColE1 family together with pECA1039, a plasmid isolated from *Pectobacterium atrosepticum *[24]. pHW42 did not fall into this subgroup and was more related to other ColE1-like plasmids (Fig. 2A). Not only the replication regions but also the multimer resolution sites (*mrs*) were closely related in all ColE1-like plasmids of *Rahnella*. In a phylogenetic tree based on *mrs *sites (Fig. 2B) most plasmids isolated from *Rahnella *formed a cluster similar to the RNA II tree, confirming that they form a separate class within the ColE1 family.

It might be thought surprising that all multimer resolution sites of plasmids depicted in Fig. 1 are in the same orientation with respect to the replication origin (*oriV*). This is also true for all ColE1-like plasmids in Fig. 2A. The explanation for this observation may lie in the intimate association of replication control and multimer resolution in the stable maintenance of ColE1-like plasmids. Because all of the ColE1 replication origins in a cell function independently, plasmid dimers (which have two origins) replicate twice as often as monomers. As a result, dimers accumulate rapidly and clonally in a process known as the dimer catastrophe [25]. RNAI-RNAII copy number control counts origins rather than plasmids, so a dimer is not differentiated from two monomers. Consequently the copy number (i.e the number of independent molecules) of dimers is approximately half that of monomers. ColE1 lacks active partition, so plasmid stability requires the maintenance of a high copy number. As a result the copy number depression caused by dimer accumulation causes plasmid instability [26]. One part of the solution to this problem is the resolution of dimers or higher multimers to monomers by site-specific recombination. The multimer resolution site of ColE1 (designated *cer*, for ColE1 resolution) contains binding sites for the host-encoded recombinase XerCD and the accessory protein ArgR (Fig. 2C). They act together with PepA (whose binding site is less clearly defined) to convert dimers to monomers by site-specific recombination [27-30]. Conserved A-T tracts phased at approximately 10.5 bp intervals facilitate the curvature of the region between the ArgR and XerC/XerD binding sites, which is thought to be beneficial for recombination complex formation [31,32]. These sequence elements are conserved in the *mrs *sites of the ColE1-like plasmids (Fig. 2C).

Multimer resolution is necessary but not sufficient to combat the threat of the dimer catastrophe. A checkpoint, mediated by the small regulatory transcript Rcd, ensures that the cell does not divide before multimers have been resolved completely to monomers [33]. Rcd binds to the enzyme tryptophanase, stimulating the production of indole which inhibits cell division by an unknown mechanism [34]. Rcd is expressed from the P_*cer *_promoter within *cer*. P_*cer *_is active in plasmid multimers but is repressed in monomers by FIS and XerCD [35]. A FIS binding site important for regulation of P_*cer *_has been mapped recently [35] (Fig. 2C).

As stated above, we observed that the multimer resolution sites of the ColE1-like plasmids were in the same orientation with respect to the replication origin (Fig. 1). In other words RNAII and *rcd *are invariably transcribed in the same direction. A possible explanation could lie in the complex regulation of P_*cer*_. FIS is required for high fidelity repression of the promoter in plasmid monomers but it is the lifting of XerCD-mediated repression in plasmid dimers which is thought to induce synthesis of Rcd and the inhibition of cell division [35]. The main evidence supporting this hypothesis is that, while the mutational inactivation of either XerC or XerD in a cell containing plasmid monomers gave a substantial increase in Rcd expression, there was no induction of Rcd expression when ArgR or PepA was inactivated [35]. RNAII read-through transcription entering *cer *(or the *mrs *on related plasmids) would first displace ArgR/PepA which will ensure that P_*cer *_remains inactive. If, however, *cer *was in the opposite orientation, transcription might displace XerCD, inducing transient expression of Rcd from plasmid monomers. A similar argument can be made for the progress of the replication fork through *cer*. In the existing orientation the fork will displace ArgR before XerCD, thus ensuring that P_*cer *_remains repressed during replication. Moreover, active P_*cer *_facing in the opposite direction might transiently stall the replication fork, since active promoters can act as replication barriers [36,37].

In addition to the replication unit and the *mrs *all sequenced ColE1-like plasmids possessed a conserved open reading frame with homology to *excI *of ColE1 (Fig. 1 and Additional file 1). ExcI was originally believed to mediate entry exclusion of homologous plasmids [38] but later it was convincingly shown that *mbeD *exhibits this activity [39]. Therefore the function of ExcI remains unknown.

In addition to these general features most ColE1-like plasmids contained highly conserved regions as indicated in Fig. 1. Clearly these plasmids show a highly mosaic structure. Since pHW114A and pHW114B reside in the same strain, their similarity can be potentially explained by recent recombination events in their host. However, the structures of the other plasmids argue strongly for frequent horizontal transfer within *Rahnella *and between *Rahnella *and *Pectobacterium*, the host of pECA1039. Interestingly, none of the ColE1-like plasmids from *Rahnella *possessed any known mobilisation system.

### pHW66 is a ColE2-like plasmid

**Figure 3:**
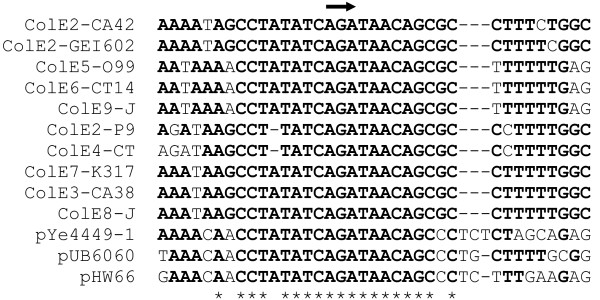
**ColE2 origins of replication**. The thick arrow indicates the primer RNA and direction of replication in ColE2-P9. Further codes as in Fig. 2.

pHW66, like the ColE1-family plasmids, showed a hybrid structure. It possessed a ColE2-like replication system composed of a *repA *gene encoding the replication protein and a conserved nucleotide sequence that might function as *oriV *(Fig. 3). While the replication origins of ColE2-like plasmids are usually located immediately downstream of the *repA *gene [40], the putative *oriV *of pHW66 was separated from *repA *by an insertion of more than 2 kb with an unusually low G+C content of 36% (the host strain of pHW66 has a G+C content of 51.8 ± 0.6%; Table 1). Interestingly, this insert comprised two genes that seemed to be cistronic with *repA*. ORF2 showed distant similarity to a putative ATPase from *Shewanella woodyi *and ORF3 was weakly homologous to a hypothetical protein from *Lyngbya sp*. (Additional file 1). Whether these genes have a role in plasmid replication or maintenance cannot be predicted. An insert of low G+C content adjacent to *repA *has also been described for the ColE2-like plasmid pUB6060 [41] but the inserts of pHW66 and pUB6060 are distinct. Another module found on pHW66 was a mobilisation system of the ColE1-superfamily composed of a conserved transfer origin (*oriT*) and 4 genes: *mobA*, *mobB*, *mobC *and *mobD *[42]. Close homologues of these genes were present on pUB6060, highlighting the close relationship between pHW66 and pUB6060. It is also interesting to note that neither pHW66 nor pUB6060 possessed a XerCD-type multimer resolution system, although this type is frequent among ColE2-like plasmids [40]. The last module was located downstream of the mobilisation system and consisted of two open reading frames with remarkable homology to two consecutive genes of unknown function in the chromosome of *Erwinia tasmaniensis *Et1/99 [43]. The significance of this will be discussed below.

### Plasmids sharing homology to rolling circle replicons

**Figure 4:**
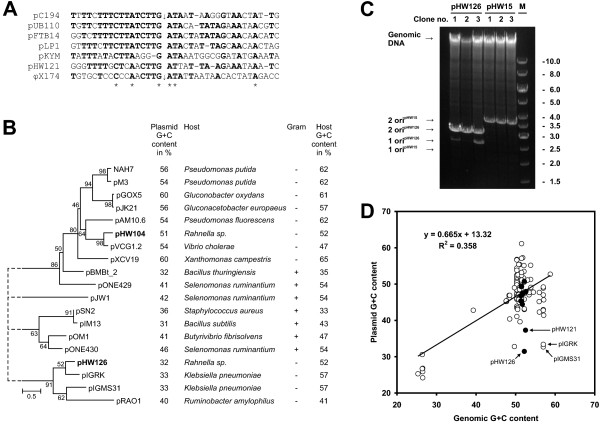
**pHW121, pHW104 and pHW126 belong to different classes of plasmids replicating by the rolling circle mechanism**. (A) A stretch upstream of the pHW121 *repA *gene is similar to replication origins of pC191/pUB110-family plasmids and the *E. coli *bacteriophage öX174. The experimentally determined cleavage sites of pC194 and öX174 are indicated by vertical arrows. (B) pHW104 and pHW126 are members of poorly characterised families of rolling circle plasmids. The G+C contents calculated for pAM10.6 and pM3 are based on partial sequences. (C) Evidence that pHW126 replicates via the rolling circle mechanism. Constructs containing two origins of replication of pHW126 and, as control, pHW15 were grown *E. coli *INVαF' for 40 generations. Subsequently DNA was isolated and analysed by restriction digestion with *Hin*dIII (similar results were obtained for digests with *Sal*I; data not shown). The expected positions of constructs containing one or two origins are indicated by arrows. The deletion of the second origin was confirmed by sequencing (data not shown). The size of the marker bands is given in kb. (D) G+C contents of small plasmids and their hosts are correlated. The trendline was calculated from 124 enterobacterial plasmid sequences retrieved from the Genome Project Database http://www.ncbi.nlm.nih.gov/genomes. For strains with unavailable genomic G+C contents the mean value of the species according to Bergey's Manual of Systematic Bacteriology [59] was used. Plasmids from *Rahnella *are shown as filled circles while plasmids from other Enterobacteriaceae are shown as open circles.

While the plasmids described above exhibited clear homology to previously classified plasmids, database searches with pHW121 retrieved only distantly-related sequences. The translated amino acid sequence of the largest ORF of pHW121 was 19%, 17% and 16% identical to replication proteins of pZMO1, pCA2.4 and pUB110, respectively (Additional file 1). Importantly, the metal binding domain showed the typical signature HUHxLUxV and the catalytic domain contained the conserved Tyr residue involved in the nucleophilic attack on the plasmid DNA at initiation of replication [44], identifying *orf1 *as *repA *and pHW121 as a member of the pC194/pUB110 family. A sequence was present upstream of *repA *that might function as *oriV *(Fig. 4A). Interestingly, the putative *oriV *was preceded by 16 perfect and 1 imperfect direct repeats of the sequence GGGTTTT. Such a motif has not been described so far for any pC194/pUB110-like plasmid. In addition, pHW121 possessed a putative mobilisation protein of the MOB_*Q *_family. Although the homology was low, the typical motifs were present [42]. Due to a lack of conservation no putative *oriT *could be identified. ORF3 of pHW121 was similar to ImcC of *Legionella pneumophila*. Several genes of the imc/dot complex are essential for the ability of *L. pneumophila *to survive in macrophages during lung infection such as Legionnaires' disease. However, no function has so far been attributed to ImcC [45].

**Figure 5:**
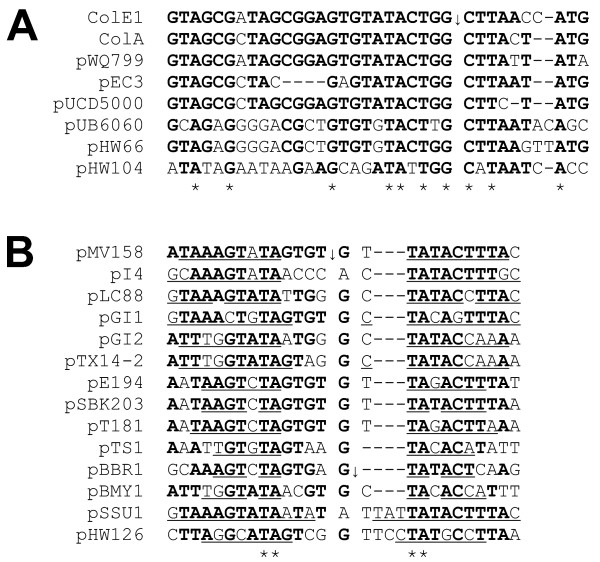
**Alignments of transfer origin (*oriT*) *nic *sites of the ColE1-superfamily (A) and the pMV158-superfamily (B)**. Experimentally determined *nic*-cleavage sites are indicated by vertical arrows. Inverted repeats involved in formation of a stem-loop-stem structure are underlined. Other codes as in Fig. 2.

pHW104 showed similarity to members of a poorly studied plasmid family (Fig. 4B). A 298 amino acid protein of pHW104 showed more than 70% identity to the putative replication protein of pVCG1.2 and 22.5% identity to RepA from pAM10.6. The involvement of the latter in replication has been proven experimentally [46]. In addition pHW104 comprised a ColE1-type mobilisation system (Fig. 5A) and two open reading frames of unknown function.

pHW126, the smallest plasmid found in the genus *Rahnella*, belonged to a novel, yet uncharacterised class of plasmids. It consisted of only 2886 bp and possessed two ORFs. ORF1 showed similarity to relaxases of the pMV158-superfamily mediating plasmid mobilisation. The characteristic motif HxDExxPHxH, as well as an invariant Arg residue in the N-terminus, were present [42] and a putative *oriT *could be identified approximately 100 bp upstream of *orf1 *(Fig. 5B). Thus *orf1 *was named *mob*. BLAST and FASTA searches with the translated amino acid sequence of ORF2 identified homologous sequences from two uncharacterised plasmids from *Klebsiella pneumoniae*, pIGRK and pIGMS31, and one plasmid from *Ruminobacter amylophilus*, pRAO1 (55.8%, 26.1%, and 22.7% identity, respectively). Iterated PSI-BLAST searches with ORF2 from pHW126 as well as with Rep from pHW104 retrieved sequences of replication proteins from pSN2-like plasmids and pJW1, indicating that all these plasmids might form a super-family (Fig. 4B). However, the Rep sequence identity between members of different clades shown in Fig. 4B was around 10% in pair wise alignments and only two amino acids are invariant in all replication proteins of the plasmids analysed (Additional file 2). A final decision whether these plasmids are members of a common super-family is not possible.

The very weak similarity of pHW126 to well characterised plasmids raised the question whether pHW126 should be classified as a rolling circle plasmid. However, we observed that increasing the size of pHW126 to more than 5 kb by insertion of foreign DNA fragments rendered this plasmid unstable (data not shown) which is a common phenomenon for rolling circle vectors [47]. To provide further experimental evidence a construct containing the *rep *gene and two copies of the upstream sequences in tandem repeat was generated. These upstream sequences are presumed to contain the origin of replication which is usually located 5' of the *rep *gene in rolling circle plasmids. This construct was transformed into the *recA*^- ^strain *E. coli *INVα F' and independent clones were grown for 40 generations. Plasmid DNA prepared from these cultures showed two bands after linearisation with restriction enzymes (Fig. 4C). The larger band of approximately 3.1 kb corresponded to the introduced plasmid. The smaller band, present in variable amounts, had a size of approximately 2.7 kb, consistent with the loss of one copy of the origin of replication. Frequent deletion of one replication origin is evidence for a rolling circle replication mechanism, because replication initiated at the second origin may terminate at the first. This causes that the part of the plasmid between the two origins to be deleted [47]. As a control a similar construct containing two copies of the *ori *from pHW15 (a ColE1 like plasmid replicating by a theta mechanism [6]), was tested in the same way. This construct maintained both origins as indicated by presence of only one band with a size of 3.7 kb (deletion of one *ori *would have reduced the size to 2.5 kb). These data provide convincing evidence that pHW126 replicates by the rolling circle replication mechanism, and that the origin of replication is located upstream of the *rep *gene.

Both pHW121 and pHW126 showed strikingly low G+C contents of only 37.3% and 31.5%, respectively. Usually the G+C contents of plasmids are correlated with the chromosomal G+C contents of their hosts (Fig. 4B and 4D). pHW121 as well as pHW126 and its close homologues pIGRK, pIGMS31 and pRAO1 clearly deviate from this rule. Many rolling circle plasmids isolated from Gram positive bacteria have a low G+C content [48-50] (Fig. 4B). Thus pHW126 and its homologues might have been acquired from Gram positive bacteria. On the other hand, *Ruminobacter amylophilus*, the host species of pRAO1, has a G+C content of approximately 41%. Recently plasmids with low G+C content in their replication regions, which are distinct from pHW121 or pHW126, were isolated from soil bacteria. These plasmids could replicate in *E. coli *but their natural host might be *Acinetobacter *[51], a genus of Gram negative bacteria with a G+C content of about 40%. Also some genera of the Enterobacteriaceae, e.g. *Buchnera*, *Hamiltonella*, *Proteus *or *Moraxella *have strikingly low G+C contents. It will be interesting to see if plasmids similar to pHW126 are isolated from such genera or from Gram positive microorganisms in the future.

### Evidence for horizontal exchange of genetic information between plasmids from *Rahnella *and bacterial chromosomes

Several plasmids possessed genes or regions homologous to sections of enterobacterial chromosomes (Additional file 1). The most interesting examples were parts of pHW66, which were homologous to the chromosome of *Erwinia tasmaniensis *Et/99, and a gene cluster of pHW4594 similar to an operon of *Photorhabdus luminescens *TT01. Stretches of approximately 1600 bp and 140 bp of pHW66 had identities of more than 90% to parts of the chromosome of *E. tasmaniensis *Et1/99 at the nucleotide level (Fig. 1). The 140 bp region of pHW66 was a small part of the plasmid *mobA *gene while the 1600 bp region comprised *orf5 *and 89 bp upstream of it, *orf6*, the intergenic region between *orf6 *and *repA *and the main part of *repA*. The corresponding region on the *E. tasmaniensis *chromosome had a similar architecture: two small open reading frames of unknown function and a *repA*-like gene. Interestingly, while RepA proteins encoded by ColE2-like plasmids showed a high degree of similarity from the N- to the C-terminus, the RepA-like protein of *E. tasmaniensis *Et1/99 was highly similar at the N-terminus but the last 45 amino acids were unrelated (Additional file 3). This RepA version might therefore not be functional. A BLAST search with the *E. tasmaniensis *Et1/99 region homologous to pHW66 indicated a hybrid structure: the 3' part harbouring the two ORFs was similar to other enterobacterial chromosomes, while the 5' part containing the truncated *repA *retrieved only plasmid sequences. With the full-length sequence there was no hit apart from pHW66. This region of the *E. tasmaniensis *Et1/99 chromosome might therefore be the result of a recent insertion of a part of a plasmid related to pHW66.

**Figure 6:**
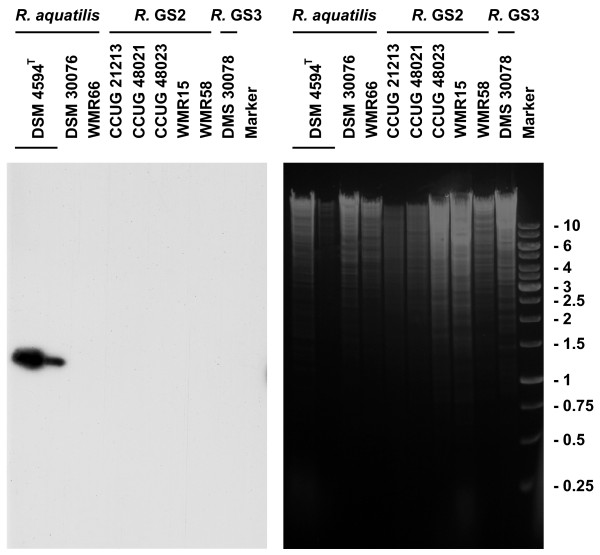
**The *orf4 orf5 orf6 *gene cluster of pHW4594 is not derived from its host**. DNA from different *Rahnella *strains was digested with *Hin*dIII (right panel) and subsequently analysed with an *orf5 orf6 *specific probe (left panel). Two different amounts of DNA were loaded of DSM 4594^T^, the host strain of pHW4596, to account for plasmid copy number (approximately 3 μg and 0.2 μg in the first and second lane, respectively). The detected band corresponded to the restriction fragment of the plasmid pHW4594 with an expected size of 1.3 kb. The same result was obtained with *Hpa*II digested DNA (data not shown). GS, genomospecies.

pHW4594 possessed a cluster of three genes, *orf4*, *orf5 *and *orf6*, that showed homology to an operon of the *P. luminescens *chromosome (Fig. 1). Although similar genes were also present in other genera, this particular arrangement could only be observed in *P. luminescens*. However, since the chromosome of *Rahnella *is not sequenced, this gene cluster of pHW4594 might also originate from the genome of its host. To test this hypothesis we investigated genomic DNA of several *Rahnella *strains by Southern blot analysis using a probe containing the main parts of *orf5 *and *orf6 *(Fig. 6). Only in the host strain of pHW4594, DSM 4594^T^, a signal could be detected which corresponded to the expected restriction fragment of the plasmid itself. Signals indicative of genomic copies of *orf5 *and *orf6 *could neither be detected in DSM 4594^T^, nor in any other strains of *Rahnella aquatilis*. Different strains of *Rahnella *genomospecies 1 and genomospecies 2 did not show any signal either. Thus, it is most likely that the *orf4 orf5 orf6 *gene cluster originates from *P. luminescens *(or another species) but not from *Rahnella*.

*Photorhabdus *is an enterobacterial symbiont of soil nematodes that infect various insects. After the nematode attacks an insect *P. luminescens *is released and produces a wide range of virulence factors ensuring rapid insect killing [52]. Recently it has been shown that *Rahnella *is the predominant species in the intestinal tract of the ghost moth *Hepialus gonggaensis *[53], indicating that *Rahnella *might frequently be present in insects. On the other hand, *E. tasmaniensis *is common on apple and pear barks and blossoms, and *Rahnella *has been isolated from apple and pear fruits [5,6,54]. Therefore, *Rahnella *seems to have overlapping habitats with *P. luminescens *and *E. tasmaniensis*, which might favour exchange of mobile genetic elements between *Rahnella *and these species.

## Conclusions

The frequency of small (less than 15 kb) plasmids is highly variable within the Enterobacteriaceae. For instance, they are extremely rare in *Citrobacter freundii *while 42% of *Escherichia coli *isolates possess at least one plasmid [23]. For the genus *Rahnella *we observed plasmid-containing isolates at a frequency of 19%, which is in the average range. ColE1-like plasmids were the predominant family, which is typical for enterobacterial genera. Most ColE1-like plasmids from *Rahnella *formed a subgroup within the ColE1 family on the basis of RNA II or *mrs*-based phylogenetic trees. The *mrs *sites of the ColE1-plasmids were arranged in a constant orientation with respect to the replication origin. Such conservation is likely to prevent inappropriate activation of the P_*cer *_promoter by read-through transcription or during replication. High-fidelity control of P_*cer *_is essential because the promoter directs expression of a small RNA that causes inhibition of cell division by stimulating indole production. The ColE1-plasmids shared extensive regions of high sequence homology in coding and in non-coding regions, indicating frequent horizontal gene transfer and recombination events among plasmids within the genus *Rahnella*. Interestingly, none of the ColE1-like plasmids found in this study possessed a mobilisation system. In contrast, the other plasmids analysed (one ColE2-like plasmid and three rolling circle plasmids) contained mobilisation genes. pHW121 is a member of the pC194/pUB110 family. pHW104 and pHW126 belong to different groups of poorly-characterised plasmids and might form a super-family with pSN2-like plasmids and pJW1. To our surprise the plasmids lacked genes which confer an obvious benefit upon their hosts. Of course some of the genes with unknown function might encode proteins with advantageous functions but at least for some of the plasmids the term "selfish DNA" seems appropriate. The best example is pHW126, the smallest plasmid found in *Rahnella*. This plasmid possessed only two ORFs, a putative replication gene and one for mobilisation. Since these coding sequences cover more than 70% of the plasmid, and additional regions are expected to function as *oriV *and *oriT*, the plasmid is simply too small to bear any gene beneficial to the host. The low G+C content of this plasmid might indicate that *Rahnella *is not its normal host. In contrast, the close similarities among the ColE1-like plasmids provided compelling evidence that *Rahnella *is their normal host. The presence of genes probably derived from *P. luminescens *on pHW4594 and stretches of the chromosome of *E. tasmaniensis *highly homologous to parts of pHW66 highlight the importance of plasmids for genetic exchange of even chromosomal sequences among different genera.

## Methods

### Media and growth conditions

*E. coli *and *Rahnella *strains were grown in MLB medium (10 g/l peptone, 5 g/l yeast extract, 5 g/l NaCl, pH 7) at 37°C and 30°C, respectively, if not otherwise indicated. When necessary, ampicillin was added to a concentration of 100 mg/l.

### Isolation and identification of *Rahnella* strains

Different types of plant materials (Table 1) were homogenised in sterile PBS and dilutions plated on Levine-EMB agar (Merck, Darmstadt, Germany). After incubation at 36°C for 48 ± 8 h dark colonies were sampled and restreaked twice on MLB plates to obtain pure cultures. Strains were classified by routine biochemical tests and partial 16S rRNA gene sequencing [6]. For amplification of the 16S rRNA gene the primer pair fD2 and rP1 was employed. The PCR product was purified with a Nucleospin Kit (Macherey-Nagel, Düren, Germany) and directly sequenced using the primers 16S-3 and 16S-5. Primer sequences are shown in Additional file 4.

### Cloning and sequencing of the plasmids

Presence of plasmids was investigated by standard alkaline lysis miniprep [55] and subsequent agarose gel electrophoresis. The strains WMR15 and WMR58 (Table 1) were used as positive and negative controls, respectively. Plasmid DNA for cloning was isolated with a Genomed plasmid midi kit and further purified by agarose gel electrophoresis. Plasmid DNA was digested with appropriate restriction enzymes and cloned into pBluescriptIIKS^+ ^(Stratagene, La Jolla, CA) cut with the same enzyme or an enzyme forming compatible ends. Both strands were sequenced by primer walking. A complete sequence for each plasmid was obtained by assembling individual reads with ContigExpress from the VectorNTI package (Invitrogen, Carlsbad, CA).

### Sequence annotation and phylogenetic analysis

Plasmid DNA sequences and predicted open reading frames were used for BLAST, PSI-BALST and FASTA databank searches at the genebank http://www.ncbi.nlm.nih.gov and ddbj http://www.ddbj.ac.jp websites. AlignX from the VectorNTI package was used to identify further less conserved or short elements e.g. *oriV*, *oriT *or *ssi *sites. The same program was employed to calculate the global identity of plasmid ORFs and sequences retrieved from databases. Phylogentic analyses were performed with MEGA4 [56]. Neighbour-joining (NJ) trees were constructed using the p-distance model for DNA and the JTT matrix for amino acid sequences. Positions with gaps were usually completely deleted except for alignments containing highly diverse sequences, where pair wise deletion was chosen. Bootstrap values were calculated from 1000 replicates and indicated at the corresponding nodes. Almost identical tree topologies were obtained with other methods (minimum evolution and UPGMA) and models (Poisson correction, PAM). G+C contents of plasmids were calculated using ARTEMIS 10 [57].

### Detection of ori deletion

pHW126 was digested with *Bgl*II and *Hin*dIII and the 1463 bp fragment containing the putative *rep *gene and the upstream intergenic sequences cloned into pBKanT [6] linearised with *Bam*HI/*Hin*dIII. The resulting construct, designated pB126ΔBH, was digested with *SpeI*, the 446 bp fragment isolated and cloned into the same construct digested with *Xba*I which led to construct pB126-2ori. This construct was used to assay replication origin deletion: pB126-2ori was digested with *Sal*I and the fragment containing the Kan^R ^gene and the pHW126 sequences isolated by agarose gel electrophoresis. The purified DNA was diluted to a concentration of 1 ng/μl and self-circularised by incubation with 1 U T4 ligase for 1 h at room temperature in a total reaction volume of 20 μl leading to pHW126-2ori. After transformation into *E. coli *INVα F' the cells were plated on MLB-kanamycin (30 μg/ml) plates and incubated overnight at 37°C. Three individual colonies were transferred completely to 100 ml MLB-Kan medium each and grown overnight. Plasmid DNA was isolated from these cultures using a Genomed plasmid midi kit as recommended by the manufacturer. Formation of plasmids containing only one pHW126 origin of replication was observed by agaraose gel electrophoresis after digestion with *Hin*dIII or *Sal*I. For confirmation, both bands were cut out, extracted with a Macherey-Nagel gel extraction kit and used as a template for PCR amplification with the primer pair pHW126-11/Kan rev. The amplification product was cleaned and directly sequenced employing the same primers as used for PCR. As a control pHW15-2ori, which possesses two pHW15 origins of replication in tandem repeat, was tested in the same way. pB15In(*Nsi*I) was constructed by inserting pHW15 [6] linearised with *Nsi*I into pBKanT. Subsequently, this construct was linearised with *Hin*dIII and *Pst*I and ligated with the 1218 bp fragment obtained by digesting pBKanT-pHW15Δ(ORF1+2+3) [6] with *Hin*dIII and *Nsi*I. This led to construct pB15-2ori which was finally digested with *Sal*I and self-circularised to obtain pHW15-2ori.

### Southern blot analysis

Approximately 3 μg genomic DNA were digested with an appropriate restriction enzyme and separated by agarose gel electrophoresis. After denaturation with 0.5 M NaOH, neutralisation with 5× TBE and equilibration with 1× TBE the DNA was transferred to a Hybond-N^+ ^membrane (GE Healthcare, Buckinghamshire, UK) by semi-dry electroblotting using 1× TBE as transfer buffer. Cross linking was achieved by irradiation with 120 mJ/cm^2 ^UV of 254 nm. Subsequently, the membrane was pre-hybridised with Church buffer [58] containing 100 μg/ml freshly denaturated herring sperm DNA. The probe was prepared by PCR: a 50 μl reaction contained 1 U GoTaq (Promega, Madison, WI), 10 μl 5× buffer containing Mg^2+^, 1 ng pHW4594 as template, 1 μl primer mix (pHW4594-fwd/pHW4595-rev; each 5 μM), 1 μl nucleotide mix (0.5 mM each of dATP, dGTP and dTTP and 0.05 mM dCTP) and 30 μCi [α-^32^P]-dCTP (3000 Ci/mmol; PerkinElmer, Waltham, MA). After an initial denaturation step at 94°C for 5 min 35 cycles of 94°C for 30 sec, 50°C for 1 min and 72°C for 2 min were performed prior a final extension step at 72°C for 10 min. The denaturated amplicon (95°C, 10 min) was added to the blocked membrane and hybridised for 18 h at 60°C. The membrane was washed 5 times with 0.05% SDS in 1× SSC [51] at 60°C and once with distilled water at room temperature. Signals were detected by autoradiography.

### Determination of genomic G+C contents

The genomic DNA G+C contents of selected strains were determined by HPLC analysis as described previously [6].

### Nucleotide sequence accession numbers

Plasmids sequences obtained in this study were deposited in the EMBL nucleotide sequence database with the following accession numbers: [EMBL:FN429021], pHW42; [EMBL:FN429022], pHW114A; [EMBL:FN429023], pHW114B; [EMBL:FN429024], pHW120; [EMBL:FN429025], pHW4594; [EMBL:FN429026], pHW30076; [EMBL:FN429027], pHW66; [EMBL:FN429028], pHW121; [EMBL:FN429029], pHW104; [EMBL:FN429030], pHW126. Accession numbers retrieved from databases are listed in Additional file 5.

## Authors' contributions

WR conceived the study and was involved in all stages of experimental work and data analysis and drafted the manuscript. EP participated in strain isolation and manuscript preparation. MK participated in database searches and sequence annotation. DKS interpreted the results regarding the multimer resolution sites. BP participated in data analysis and helped to draft the manuscript. All authors read and approved the final manuscript.

## Supplementary Material

Additional file 1 **Annotation of the open reading frames**. A table with annotation details of the open reading frames of all plasmids isolated in this study is shown. Click here for file

Additional file 2 **Alignment of replication proteins**. The data provide an alignment of the replication proteins of pHW104, pHW126 and related plasmids. Click here for file

Additional file 3 **The RepA-like protein of the *E. tasmaniensis *Et/99 chromosome diverges at its C-terminus from plasmid RepA proteins**. The data provide an alignment of the RepA sequences of pHW66, pYe4449-1 and pUB6060 and the RepA-like gene of the *E. tasmaniensis *Et1/99 chromosome. Click here for file

Additional file 4 **Primers used in this study**. The data provide the sequences of primers used in this study. Click here for file

Additional file 5 **Accession numbers of sequences retrieved from databases**. This table provides the accession numbers of sequences retrieved from databases and used for construction of phylogenetic trees and alignments. Click here for file
