# Supplementary material for: Frequency and diversity of small cryptic plasmids in the genus Rahnella
Source: BMC Microbiol. 2010 Feb 19;10:56. doi: 10.1186/1471-2180-10-56 (PMC2831885; doi:10.1186/1471-2180-10-56)
Supplement: Additional file 1 — Annotation of the open reading frames. A table with annotation details of the open reading frames of all plasmids isolated in this study is shown. [file 1471-2180-10-56-S1.PDF]

Additional file 1: Annotation of the open reading frames

Name	Significant BLAST hit (organism / plasmid)	Accession no.	% Id[a]	Proposed function	Reference
pHW120					
ORF1	Excl1 (*Klebsiella pneumoniae* / pKpn114)	YP_002333284	58.4	Unknown	Yamada et al. 1995. J. Bacteriol. 177, 6064–6068.
ORF2	VapI (*Pectobacterium atrosepticum* / pECA1039)	YP_002429178	76.9	Virulence-associated protein; antidote protein to toxin-antitoxin system	Fineran et al., 2009. Proc. Natl. Acad. Sci. USA 106, 894-899.
ORF3	Phosphate taxis regulatory protein (*Enterobacter cloacae* / pEC01)	YP_002290993	47.2	Transcriptional regulator XRE family	Yang et al., 2004. Microbes Environ. 19, 45-52.
ORF4	Hypothetical protein (*Rhizobium leguminosarum* / pRL11)	YP_771498	36.8	Unknown	Young et al., 2006. Genome Biol. 7, R34.
pHW4594					
ORF1	Excl1 (*Klebsiella pneumoniae* / pKpn114)	YP_002333284	58.4	Unknown	Yamada et al., 1995. J. Bacteriol. 177, 6064–6068.
ORF2	Hypothetical protein (*Photorhabdus luminescens*)	NP_931308	63.0	Unknown	Duchaud et al., 2003. Nat. Biotechnol. 21, 1307-1313.
ORF3	Hypothetical protein (*Pectobacterium atrosepticum* / pECA1039)	ACK87017	95.8	Unknown	Fineran et al., 2009. Proc. Natl. Acad. Sci. USA 106, 894-899.
ORF4	Hypothetical protein (*Photorhabdus luminescens*)	NP_928372	50.7	Unknown	Duchaud et al., 2003. Nat. Biotechnol. 21, 1307-1313.
ORF5	Hypothetical protein (*Photorhabdus luminescens*)	NP_928371	60.1	Unknown	Duchaud et al., 2003. Nat. Biotechnol. 21, 1307-1313.
ORF6	Hypothetical protein (*Photorhabdus luminescens*)	NP_928370	33.3	Unknown	Duchaud et al., 2003. Nat. Biotechnol. 21, 1307-1313.
pHW30076					
Rom / ORF1	Rom (*Pectobacterium atrosepticum* / pECA1039)	YP_002429169	92.3	RNA I modulator protein	Fineran et al., 2009. Proc. Natl. Acad. Sci. USA 106, 894-899.
	Rom (*Escherichia coli* / ColE1)	NP_040367	49.2	RNA I modulator protein	Inoue and Uchida, 1991. J. Bacteriol. 173:1208–1214.
ORF2	Entry exclusion protein 1 (*Klebsiella pneumoniae* / 15S)	YP_002286825	63.3	Unknown	Gootz et al., 2008. Direct submission.
ORF3	Hypothetical protein (*Photorhabdus luminescens*)	NP_931308	63.0	Unknown	Duchaud et al., 2003. Nat. Biotechnol. 21, 1307-1313.
ORF4	No significant similarity found				
pHW114A					
Rom / ORF1	Rom (*Pectobacterium atrosepticum* / pECA1039)	YP_002429169	81.5	RNA I modulator protein	Fineran et al., 2009. Proc. Natl. Acad. Sci. USA 106, 894-899.
	Rom (*Escherichia coli* / ColE1)	NP_040367	47.7	RNA I modulator protein	Inoue and Uchida, 1991. J. Bacteriol. 173:1208–1214.
ORF2	Entry exclusion protein 1 (*Klebsiella pneumoniae* / 15S)	YP_002286825	61.7	Unknown	Gootz et al., 2008. Direct submission.
ORF3	VapI (*Pectobacterium atrosepticum* / pECA1039)	YP_002429178	79.6	Virulence-associated protein; antidote protein to toxin-antitoxin system	Fineran et al., 2009. Proc. Natl. Acad. Sci. USA 106, 894-899.
ORF4	Hypothetical protein (*Acinetobacter sp.* / pRAY)	NP_049454	44.0	Unknown	Segal and Elisha, 1999. Plasmid 42, 60-66.
ORF5	Hypothetical protein (*Shewanella baltica*)	YP_001364982	28.1	Unknown	Copeland et al., 2007. Direct submission.
ORF6	No significant similarity found				
pHW114B					
Rom / ORF1	Rom (*Pectobacterium atrosepticum* / pECA1039)	YP_002429169	95.4	RNA I modulator protein	Fineran et al., 2009. Proc. Natl. Acad. Sci. USA 106, 894-899.
ORF2	ExcI (*Pectobacterium atrosepticum* / pECA1039)	YP_002429168	91.0	unknown	Fineran et al., 2009. Proc. Natl. Acad. Sci. USA 106, 894-899.
ORF3	VapI (*Pectobacterium atrosepticum* / pECA1039)	YP_002429178	80.6	Virulence-associated protein; antidote protein to toxin-antitoxin system	Fineran et al., 2009. Proc. Natl. Acad. Sci. USA 106, 894-899.
ORF4	No significant similarity found				
ORF5	No significant similarity found				
ORF6	Hypothetical protein (*Pectobacterium carotovorum*)	ZP_03833682	82.7	unknown	Glasner et al., 2008. Mol. Plant. Microbe Interact. 21,1549-1560.
ORF7	Putative SinR-like protein (*Yersinia pseudotuberculosis*)	YP_001874505	52.2	unknown	Copeland et al., 2008. Direct submission.
ORF8	Hypothetical protein (*Pasteurella multocida*)	NP_246021	12.5	unknown	May et al., 2001. Proc. Natl. Acad. Sci. USA 98, 3460-3465.
ORF9	Hypothetical protein PM1084 (*Pseudomonas fluorescens*)	YP_002871238	12.0	unknown	Lucas et al., 2009. Direct submission.

Name	Significant BLAST hit (organism / plasmid)	Accession no.	% Id	Proposed function	Reference
pHW42					
ORF1	Entry exclusion protein 1 (*Klebsiella pneumoniae* / 15S)	YP_002286825	62.5	Unknown	Yamada et al., 1995. J. Bacteriol. 177, 6064–6068.
ORF2	VapI (*Pectobacterium atrosepticum* / pECA1039)	YP_002429178	70.7	Virulence-associated protein; antidote protein to toxin-antitoxin system	Fineran et al., 2009. Proc. Natl. Acad. Sci. USA 106, 894-899.
ORF3	No significant similarity found				
ORF4	No significant similarity found				
ORF5	Hypothetical protein (*Clostridium perfringens*)	ZP_02641501	19.6	Unknown	Paulsen and Sebastian, 2007. Direct submission.
pHW66					
MobC / ORF1	MobC (*Yersinia enterocolitica* / pYe4449-1)	YP_002643114	98.1	Plasmid mobilisation	Lepka et al., 2009. Direct submission.
	MobC (*Plesiomonas shigelloides* / pUB6060)	CAB56514	74.3	Plasmid mobilisation	Avison et al., 2001. Plasmid 45, 88-100.
	MbeC (*Escherichia coli* / ColE1)	NP_040368	40.4	Plasmid mobilisation	Francia et al., 2004. FEMS Microbiol Rev 28: 79–100.
MobA / ORF2	MobA (*Yersinia enterocolitica* / pYe4449-1)	YP_002643113	74.8	Plasmid mobilisation	Lepka et al., 2009. Direct submission.
	MobA (*Plesiomonas shigelloides* / pUB6060)	CAB56515	53.8	Plasmid mobilisation	Avison et al., 2001. Plasmid 45, 88-100.
	MbeA (*Escherichia coli* / ColE1)	NP_040369	40.1	Plasmid mobilisation	Francia et al., 2004. FEMS Microbiol Rev 28: 79–100.
MobB / ORF3	MobB (*Plesiomonas shigelloides* / pUB6060)	CAB56516	39.1	Plasmid mobilisation	Avison et al., 2001. Plasmid 45, 88-100.
	MbeB (*Escherichia coli* / ColE1)	NP_040371	21.8	Plasmid mobilisation	Francia et al., 2004. FEMS Microbiol Rev 28: 79–100.
MobD / ORF3	Putative mobilization protein (*Plesiomonas shigelloides* / pUB6060)	CAB56517	59.0	Plasmid mobilisation	Avison et al., 2001. Plasmid 45, 88-100.
	MbeD (*Escherichia coli* / ColE1)	NP_040372	38.5	Plasmid mobilisation	Francia et al., 2004. FEMS Microbiol Rev 28: 79–100.
ORF5	Hypothetical protein (*Erwinia tasmaniensis*)	YP_001909146	90.0	Unknown	Kube et al., 2008. Environ. Microbiol. 10, 2211-2222.
ORF6	Predicted transcriptional regulator (*Erwinia tasmaniensis*)	YP_001909145	98.9	Transcriptional regulator XRE family	Kube et al., 2008. Environ. Microbiol. 10, 2211-2222.
RepA / ORF7	RepA (Klebsiella sp. / pGD2)	NP_620615	76.2	Initiation of replication	Yoo et al., 2001. J. Biochem. Mol. Biol. 34, 584-589.
	RepA (*Plesiomonas shigelloides* / pUB6060)	CAB56518	70.6	Initiation of replication	Avison et al., 2001. Plasmid 45, 88-100.
ORF8	ATPase (*Shewanella woodyi*)	YP_001759243	25.1	Unknown	Copeland et al., 2008. Direct submission.
ORF9	Hypothetical protein (*Lyngbya sp.*)	ZP_01619878	22.6	Unknown	Stal et al., 2006. Direct submission.
pHW121					
ORF1	No significant similarity found				
ORF2	No significant similarity found				
ORF3	IcmC protein (*Legionella pneumophila*)	AAS91917	17.3	Unknown	Morozova et al., 2004. Plasmid 51, 127-147.
Rep / ORF4	Replication initiation protein (*Zymomonas mobilis* / pZMO1)	YP_002274345	19.2	Initiation of replication	Arvanitis et al., 2000. Plasmid 44, 127-137.
	RepA (*Synechocystis sp.* / pCA2.4)	AAA02970	17.0	Initiation of replication	Yang and McFadden, 1993. J. Bacteriol. 175, 3981-3991.
	Rep (*Staphylococcus aureus* / pUB110)	NP_040434	16.0	Initiation of replication	McKenzie at al., 1986. Plasmid 15, 93-103.
Mob / ORF5	MobB (*Bifidobacterium longum* / pKJ36)	NP_072178	18.6	Plasmid mobilisation	Francia et al., 2004. FEMS Microbiol Rev 28: 79–100.
ORF6	No significant similarity found				
pHW104					
Rep / ORF1	Plasmid replication initiation protein (*Vibrio cholerae* / pVCG1.2)	YP_001966398	72.2	Initiation of replication	Zhang and Gu, 2008. Direct submission.
	RepA (*Pseudomonas fluorescens* / pAM10.6)	AAG23805	22.5	Initiation of replication	Peters et al., 2001. Plasmid 46, 25-36.
ORF2	Hypothetical protein (*Hamiltonella defensa*)	YP_002923099	73.1	Unknown	Degnan and Moran, 2009. Direct submission.
ORF3	No significant similarity found				
MobA / ORF4	MbeA (*Escherichia coli* / ColE1)	NP_040369	29.4	Plasmid mobilisation	Francia et al., 2004. FEMS Microbiol Rev 28: 79–100.
MobB / ORF5	MbdB (*Escherichia coli* / pColD-157)	CAA71438	30.1	Plasmid mobilisation	Hofinger et al., 1998.
	MbeB (*Escherichia coli* / ColE1)	NP_040371	19.7	Plasmid mobilisation	Francia et al., 2004. FEMS Microbiol Rev 28: 79–100.

Name	Significant BLAST hit (organism / plasmid)	Accession no.	% Id	Proposed function	Reference
pHW104 (continued)					
MobC / ORF6	MobC (*Pasteurella multocida* / pB1002)	ABZ82545	27.3	Plasmid mobilisation	San Millan and Gonzalez-Zorn, 2009. Direct submission.
	MbeC (*Escherichia coli* / ColE1)	NP_040368	25.4	Plasmid mobilisation	Francia et al., 2004. FEMS Microbiol Rev 28: 79–100.
pHW126					
Mob / ORF1	Mob (*Pseudomonas* syringae / pPMA4326D)	YP_025700	21.1	Plasmid mobilisation	Stavrinides and Guttman, 2004. J. Bacteriol. 186, 5101-5115.
	Pre (*Staphylococcus aureus* / pT181)	NP_040472	16.1	Plasmid mobilisation	Francia et al., 2004. FEMS Microbiol Rev 28: 79–100.
Rep / ORF2	ORF1 (*Klebsiella pneumoniae* / pIGRK)	YP_002290994	55.8	Unknown	Kaczanowski et al., 2004. Direct submission.

[a] Percent global identity at the amino acid sequence level.
