# Supplementary material for: Frequency and diversity of small cryptic plasmids in the genus Rahnella
Source: BMC Microbiol. 2010 Feb 19;10:56. doi: 10.1186/1471-2180-10-56 (PMC2831885; doi:10.1186/1471-2180-10-56)
Supplement: Additional file 2 — Alignment of replication proteins. The data provide an alignment of the replication proteins of pHW104, pHW126 and related plasmids. [file 1471-2180-10-56-S2.PDF]

```
           1                                                                                             95
NAH7       -----------------------------------------------------------------MVDDEGEVRALVQPTQR----GTWVQTERA
pM3        -----------------------------------------------------------------MANDKNEIRAYAQPAQR----GTWVQTERA
pGOX5      ---------------------------------------------------------------------MKSKQVGLTTAPN----GTWVQVERA
pJK21      ---------------------------------------------------------------------MKPKHVGLTTAPN----GTWVQVERA
pAM10.6    ----------------------------------------------------------------MFTIPHGYVHLKQIKKDP----ADFAMLTRG
pHW104     --------------------------------------MAHDENTDELVERNK-RLNELLQNTAAQLQEERRLNLLTKNRVT----GGYYMMSRA
pVCG1.2    --------------------------------------MSDDKNKIETLEQQLSQARALLSHTVDTLQEERRLAAATKNRVT----GGYYMMSRA
pXCV19     -----------------------------------------------------------------------MSLPVPSSKKR----IEYVQLYRS
pBMBt_2    -------------------MFKIYERSKNKTMKNENETDIKISKEEFMKIIELTGLPISHIQKLIDLEKAEQEEAKRKELEKKN--PPFVQLYKS
pONE429    ----------------------------------------MKNLSDAQKIIAQGNREETKEERLQRLEEEERAEIKQAEGAKKSPYFNFLQVNQA
pJW1       ----------------------------------------------MENLDTKVIPLPTERECTVVLGADGEVKGYINKPRFNKLGREYFVGFKS
pSN2       -------------------------------------------------MKERYGTVYKGSQRLIDEESGEVIEVDKLYRKQ----TSGNFVKAY
pIM13      -------------------------------------------------MKERYGTVYKGSQRLIDEESGEVIEVDKLYRKQ----TSGNFVKAY
pOM1       -----------------------------------------LAKNELSQHVTTKKVKAIGTETYINVATGEAEEFQVTKIEE----RDFNFTKVW
pONE430    --------------------------------------------MDKKPVTTRKKVKVTGTETYINQSTGEIKEMQVIDIEE----RDFNFHKVW
pHW126     ----------------MNIQQILNDSLNISYENFNDYLNQYDLSPEEKVAAFTFYHENVKQKKAITISAEKTNNYKSIADKV----EYVHLFPKN
pIGKR      ----------------MDIGNILNESLSIDYEKLDLFLEKYDLTPEQKVAVYEFHAKAYKKNKTLVISETKENKFKSISEGV----EYVHLFPKN
pIGMS31    MKKNKLVNKENYSILETLPEDPLFENKSTLEIDLNQFDLFNRIANETVEELIIKEVNDPNDRSDKSNGVNLNAKVYVEKEKKTSLKKDFVITFVD
pRAO1      ----------------------------------------------------------------------MDISVNVARQRIKPDQEFIFYFLTN
Consensus                                                                             V             F  V

           96                                                                                            190
NAH7       GHEAWAALIAQAPRAGQLMHIIVQHMDK-SGALIVSQSTLAKMMDTSIATTKRAISVLAKHNWIQTISVGGQRGGTLAYVVNSRIAWADKREN-L
pM3        GHEAWAALTAQAPRAAQLMHILVQHMDK-QGALIISQATLAKLMETSVATTKRAIAILTKHNWIQTISVGGQRGGTLAYVVNSRIAWADKRDN-L
pGOX5      AMERWSKLAVSNPRAAAVMMLMTSQMGR-NNALVASQATLAKMAGCGLNTLKRALSVLREGNWIEVRQIG-PTGTACAYVVNDRVAWSGNRDG-I
pJK21      AMERWSKLAVSNPRAAAVMMLMTSQMGR-NNALVASQATLAKMAGCGLNTLKRALSVLREGNWIEVRQIG-PTGTACAYIVNDRVAWSGNRDG-I
pAM10.6    YIRDIRELSRRSPSAFQVFMLLTERMNK-TNAIVISQSTLCQILSYGRTAIHNAIRLLESERWLQIVKIG----TANGYVINSKVVWRDHSGK--
pHW104     AEKNLRALQKENATAALVFSVIRENMQIGTNAVTISNPVLAKILGKSARTVARATKHLAQHAYVQIVKVG----NTNTYIVNEQVAFAGSVG--Q
pVCG1.2    AEKNLRALQKANPAAALVFSVIRENMQIGTNAVAISNTAFCKIIGKSRATVTRAIKHLSDHHYVQIVKIG----TTNTYVVNEQVAFAGSAG--Q
pXCV19     SMKALRQLALEAPTAHAVLYVLMERINE-RNALVASYATLAKLTGKSRATITRALAELRTRNYIEMVKAG----NVSVIVVNKRVAWATETALRG
pBMBt_2    HMKEIRWLITNHHLSSNILFFFLENMNN-RNVIVCSQQLLMEQFNKGRTTIHNAIKTLKEHGFISVAKIG----NANAYIINPEIAFQDSRDK-I
pONE429    NYKAEDWLMRESPPAYRLLRFIAQNMDN-YNALMCSYKVFQESLGYGRATIARAVKLLKEKNFIRIAKSG----TANIYLVNKQLYWHSYGTN-Y
pJW1       GLEGLASMDLTGEQYKVLMYLFSRLDFD--NFLKVPQKEISEKLNLHKSNVSKAIKKLAELDVIAVGPMAG---HSKTYRLNPRIAHRGLRTTKK
pSN2       IVQLISMLDMIGGKKLKIVNYILDNVHLSNNTMIATVREIAEGTNTSTKTVNTTLKILEEG-NIIKRRTG-------ALMLNPELLMRGDDQK-Q
pIM13      IVQLISMLDMIGGKKLKIVNYILDNVHLSNNTMIATTREIAKATGTSLQTVITTLKILEEG-NIIKRKTG-------VLMLNPELLMRGDDQK-Q
pOM1       IRNFVATLDLVGNQKTRLVYWIIDNLNHNN-QLICTNRLMAEETGISLATVSVTMKALQDANFLKKQANG-------VYVINPDILFKGTQKA-R
pONE430    LEHIIHSMDLIGNQKTRLAFWLINNLNRDN-VLIMTQRKIAEKTGISLETVRQTMKALMESGFLIKINSG-------AYCVNPDAVFKGGKTD-R
pHW126     LKKIIKEYNLTSNELLVTMEILDSMMSHGNMLINFSQARLCELTEINKSTMSKVFKSLRAKKVLIENENG-------NLYINSVVFMKG-LPH-K
pIGKR      LKILIKKYGLNTNELLVLTEIMESMLSHGNLLINFSQKALCELTGINKSTMCKTFKTLKQKQCLIE-KNG-------HIYLNSVIFMKG-LPH-K
pIGMS31    NLEALAKLNLKPNEFRIIVEIVKVMEYG--NLINLSQSTIAKNLNLAKSNVSYYFKNLKKK-NILVEKDG-------HVFMNSNIFSKGLAHR--
pRAO1      MDSLISDPDITKQDIAVLMKYAAKMQYG--NQISIAQADIAEDLGIDKSNVSKSVRKLTQKGVFLKERRS--------LVMNWKYLAKGNLTDFI
Consensus      IA L L    A  LM II  NM    N LI SQ  LA L G SKATV RAIK L E  WI V K G        YVVN  I WKG

           191                                                                                  279
NAH7       QYARFNARVLISSEDQADLGSD----KLKQLPTMSDGEIQLPAGPGMEPPAQESLDGVDLPDMPSIPHHGKNDV---------------
pM3        QFALFNARVLVSTEDQADLGDA----KLKQLPTMEDGDIQLPAGPGMDPPAQESLEG-MLPDMPSIPHGN-------------------
pGOX5      RYSLFSASVLLADNEQPDKDEIGLQPSLHPVPDLYPGEKQLPTGPGLPPPSQPSFDG-MEPDLPATEK---------------------
pJK21      RYSLFSAAVLLSDDEQPDKTEIGAQPPLQAIPDLYPGEKQLPTGPGLPPPSQPSFDG-MEPDLPATEK---------------------
pAM10.6    RYASFYAEVVVSESDQGRPVEDWDNIELRHVPVLHAGESVVDDGAELPPPDQQDLLPPEPHEFPRTSATHEPQAVADTRAPPTRSTGPR
pHW104     RKAVFSATVVAHECEQDEGWDQVK--KLKAIPIIFDDERPLLSEEVLPPPDQTDLDLN-------------------------------
pVCG1.2    RKAVFSATVVAHECEQEEGWDEVK--KLKAVPVIYEDERPILGCDELPPPDQQDLDLN-------------------------------
pXCV19     KLAVFDARVIVSSEEQDEPERLGKEPDLVRLPPVLVPPEMATMLEDGEDDGQGDLQL--------------------------------
pBMBt_2    KYVSFEGKILINKNENEELFKEHKFENLKVLKDEKK-----------------------------------------------------
pONE429    ARAEFGAKIIVTADEQEPEDRKQIEFDVKRQKILDVKETTCVEEEPNLFTNEQLQAI--------------------------------
pJW1       PLFSMTH----------------------------------------------------------------------------------
pSN2       KYLLLEFGNFEQEDDQKQENALSEYYSFKE-----------------------------------------------------------
pIM13      KYLLLEFGNFEQEANEID-----------------------------------------------------------------------
pOM1       LNILNQFSELGAEPQELSDEQKIQNITKTIAQLSKQLEALQSKSSVVDTEIEGQYTLLPDGSIVQKAVNKKE-----------------
pONE430    LNVLIQYRKSEQE--NTKTSEPTSEKDEPSLFNNEQLKAM-------------------------------------------------
pHW126     LFIQYREHFLKSIEYKLTEEENFDQVFDAEFIKTYESNIKKIKDKKEELEQKKKDKTLKSFNETLKTELKEAT---EENFDLIFDEQN-
pIGKR      LFMQFRDHFLNSISYKLDDEEEFEKVFDDNFIKAYEKNLKEIKKKKQQIKEKKISKALDNFEKEISKEWKEKFKDEEENFEFGFESEI-
pIGMS31    -LDEEKRKNLKSAQVEDDNFKNSF-----------------------------------------------------------------
pRAO1      KADKENSKLSKFQLVEDEE----------------------------------------------------------------------
Consensus  KY LF A VL S  EQ D         L  L
```

Additional file 2: Alignment of replication proteins. Identical amino acids are shown red, conserved green (present in at least 50% of the sequences) and amino acids with similar properties are highlighted in yellow.
