# Supplementary material for: Frequency and diversity of small cryptic plasmids in the genus Rahnella
Source: BMC Microbiol. 2010 Feb 19;10:56. doi: 10.1186/1471-2180-10-56 (PMC2831885; doi:10.1186/1471-2180-10-56)
Supplement: Additional file 3 — The RepA-like protein of the E. tasmaniensis Et/99 chromosome diverges at its C-terminus from plasmid RepA proteins. The data provide an alignment of the RepA sequences of pHW66, pYe4449-1 and pUB6060 and the RepA-like gene of the E. tasmaniensis Et1/99 chromosome. [file 1471-2180-10-56-S3.PDF]

```
                          1                                                                              80
                pUB6060   --------------MANQALTLFNDRLPHKPYFSDDLQFGVRIAGKERALLAKYIQFNQPHAMYWLCFDVDRAGAAIDWA
              pYe4449-1   MHRAKQQPAIAQARLSNQALDLFNERLPRKPYFTDELQFGVRIAGKERAILAKYIQFNQPHAMFWLGFDVDRPGAAIDWS
                  pHW66   --------------MTKPALDLFNERLPHKPYFSDDLHFGVRIAGKERAILAKYIQFNQPHAMFWLGFDVDRLGAAIDWS
E. tasmaniensis Et1/99   --------------MTNPALDLFNDRLPNKPYFSDDLHFGVRIAGKERAILAKYIQFNQPHAMFWLGFDVDRLGAAIDWS

                          81                                                                            160
                pUB6060   DLGAPAPTLTIKNPDNGHAHLLYALNIAVRTAPDGRGRLLKYAAAIENALRKKLGADAGYSGLICKNPNHLHWQITVWQP
              pYe4449-1   DRNAPAPTLTIMNPENGHAHLLYALKTAVRTAPDGKIKPLKYAAAVERALCQKLDADTGYSGLICKNPNHDHWQLAVWQP
                  pHW66   DRNAPAPTLTITNPENGHAHLLYALKTSIRTAPDGKMKPLKYAAAVENALRKKLDADVGYSGLICKNPNHGYWKIAVWQP
E. tasmaniensis Et1/99   DRNAPAPTLTITNPENGHAHLLYALKTSIRTAPDGKMKPLKYAAAVENALRKKLDADTGYSGLICKNPNHGHWKIAVWQP

                          161                                                                           240
                pUB6060   ELYTLDWLADYLDLGAANDREILPDYGLGRNCTLFDKTRKWAYRAIRQGWPEYSQWLQACIERAKAYNLQFSAPLDENEV
              pYe4449-1   ELYTLDWLADSLDLNAANDKEIVADYGLGRNCTLFDKTRKWAYRAIRQGWPQYEQWLQACYERARAYNLQFSAPLDETEV
                  pHW66   ELYTLDWLADSLDLNAANDKEIVADYGLGRNCTLFDKTRKWAYRAIRQGWPEYEQWLQACYERSSAYNLQFAMPLDESEV
E. tasmaniensis Et1/99   ELYTLDWLADSLDLNAANDKEIVADYGLGRNCTLFDKTRKWAYRAIRQGWPEYYQWLQACYERARAYNQQFATPLDENEV

                          241                                                                           320
                pUB6060   MGIAKSISKWTMVTYRSLGFDEYVKLTHSPEVQAYRGRRSKGGGRP-SIGEP-----WLALGISRRSYFRWKKLGKL---
              pYe4449-1   SGIAKSIAKWTYKNFSEANFLQYIADTHSSEIQSKRGMKSRGGGRPKIVGSP-----WLNLGISRSKWYRDFRGL-----
                  pHW66   FGVARSIAKWTSKNFSESDFIKYVVNTHISSIQSKRGKKSKGGGRKLNVNSESQKKPWIEIGISRSTYYRKKNDGKNNQV
E. tasmaniensis Et1/99   NSIAKSIAKWTSGKFSESSFLDFLLEHTSLIFNQSEESVVKVEVAPFLVILG---RNWKLVGLPILEN------------
```

Additional file 3: The RepA-like protein of the *E. tasmainensis* Et/99 chromosome diverges at its C-terminus from plasmid RepA proteins. The RepA sequences of pHW66 and pYe4449-1 were translated from the nucleotide sequence while RepA of pUB6060 and *E. tasmaninesis* Et1/99 were retrieved from databases. Colour codes as in Additional file 2.
