# Supplementary material for: Frequency and diversity of small cryptic plasmids in the genus Rahnella
Source: BMC Microbiol. 2010 Feb 19;10:56. doi: 10.1186/1471-2180-10-56 (PMC2831885; doi:10.1186/1471-2180-10-56)
Supplement: Additional file 4 — Primers used in this study. The data provide the sequences of primers used in this study. [file 1471-2180-10-56-S4.PDF]

Additional file 4: Primers used in this study.

Name	Sequence (5´-3´ direction)	Purpose[a]
fD2<br>rP1	AGAGTTTGATCATGGCTCAG<br>ACGGTTACCTTGTTACGACTT	Amplification of the 16S rRNA gene (T_A: 42°C)
16S-3<br>16S-5	ATATTGCACAATGGGCGC<br>GCCATTGTAGCACGTGTGTAG	Sequencing of the amplified 16S rRNA gene
pHW126-11<br>Kan rev	TCATAATTAAAACCTCCATT<br>GCAGACAGTTTTATTGTTCATGATG	Amplification (T_A: 50°C) and sequencing in the ori-deletion experiment
pHW4594-fwd<br>pHW4594-rev	CGTCACCAACCGTCATCG<br>GTGTGTCTTGTAAGGTGCAGAGC	Preparation of a probe for Southern blot analysis by PCR (T_A: 50°C)

[a] T_A: Annealing temperature used for PCR.
