# Supplementary material for: Frequency and diversity of small cryptic plasmids in the genus Rahnella
Source: BMC Microbiol. 2010 Feb 19;10:56. doi: 10.1186/1471-2180-10-56 (PMC2831885; doi:10.1186/1471-2180-10-56)
Supplement: Additional file 5 — Accession numbers of sequences retrieved from databases. This table provides the accession numbers of sequences retrieved from databases and used for construction of phylogenetic trees and alignments. [file 1471-2180-10-56-S5.PDF]

Additional file 5: Accession numbers of sequences retrieved from databases

Name	DNA sequence	Rep protein
Plasmids		
15S	NC_011382	
CloDEF13	NC_002119	
ColA	NC_001373	
ColE1	NC_001371	
ColE2-CA42	D30056	
ColE2-GEI602	D30057	
ColE2-P9	D30054	
ColE3-CA38	D30055	
ColE4-CT	D30059	
ColE5-O99	D30060	
ColE6-CT14	D30061	
ColE7-K317	D30062	
ColE8-J	D30063	
ColE9-J	D30064	
NAH7	NC_007926	YP_534792
p9123	NC_005324	
pAM10.6	AF269166	AAG23805
pBBR1	X66730	
pBERT	NC_001848	
pBMBt_2	NC_006821	YP_195275
pBMY1	AJ243967	
pBS512_7	NC_010672	
pC194	NC_002013	
pCol-let	NC_002487	
pE194	V01278	
pEC3	D45188	
pEC904	AY589570	
pECA1039	FJ176937	
pEI1	NC_002497	
pEsp1396	AF527822	
pFTB14	X06242	
pGI1	NC_004335	
pGI2	X13481	
pGOX5	NC_006676	YP_190452
pHW15	NC_008053	
pI4	AF300457	
pIGMS31	AY543072	AAS55464
pIGRK	AY543071	AAS55462
Plasmids		
pIM13	M13761	AAA98135
pJK21	CAA11421	AJ223503
pJW1	AF141869	AAD30138
pKlebB-k17/80	NC_002610	
pKPN6	NC_009652	
pKPN7	NC_009653	
pKYM	NC_001378	
pLC88	U31333	
pLP1	M31223	
pM3	AF078924	AAD46123
pMV158	X15669	
pOM1	L31579	AAB57762
pONE429	NC_004986	NP_862700
pONE430	AB003193	BAA24992
pPA3.0	EU092237	
pRAO1	AB022866	BAA74510
pRK2	NC_005970	
pSERF1	NC_005862	
pSKB203	U35036	
pSMS35_8	NC_010485	
pSN2	V01282	CAA24595
pSSU1	NC_002140	
pT181	NC_001393	
pTS1	NC_002650	
pTX14-2	NC_004334	
pUB110	NC_001384	
pUB6060	AJ249644	CAB56518
pUCD5000	AF022806	
pVCG1.2	NC_010899	YP_001966398
pWQ799	L39794	
pXCV19	NC_007505	YP_361513
pYe4449-1	NC_012208	
Other sequences		
φX174	J02482	
E. tasmaniensis Et1/99	NC_010694	YP_001909144
P. luminescens TT01	NC_005126	
